# Variants in *KMT2A* in Three Individuals with Previous Suspicion of 22q11.2 Deletion Syndrome

**DOI:** 10.3390/genes15020211

**Published:** 2024-02-06

**Authors:** Henrique Garcia Silveira, Carlos Eduardo Steiner, Giovana Toccoli, Luise Longo Angeloni, Júlia Lôndero Heleno, Samira Spineli-Silva, Ana Mondadori dos Santos, Társis Paiva Vieira, Maria Isabel Melaragno, Vera Lúcia Gil-da-Silva-Lopes

**Affiliations:** 1 Disciplina de Genética, Departamento de Morfologia e Genética, Universidade Federal de São Paulo (Unifesp), São Paulo 04023-062, Brazil; hsilveira@unifesp.br (H.G.S.); giovana.toccoli@unifesp.br (G.T.); melaragno.maria@unifesp.br (M.I.M.); 2 Genética Médica e Medicina Genômica, Departamento de Medicina Translacional, Faculdade de Ciências Médicas, Universidade Estadual de Campinas (Unicamp), Campinas 13083-970, Brazil; steiner@unicamp.br (C.E.S.); luisel@unicamp.br (L.L.A.); londero@unicamp.br (J.L.H.); samiraspineli@gmail.com (S.S.-S.); anamondadori.dra@gmail.com (A.M.d.S.); tpvieira@unicamp.br (T.P.V.); 3 Faculdade São Leopoldo Mandic (SLMandic), Campinas 13045-755, Brazil

**Keywords:** 22q11.2 deletion syndrome, Wiedemann–Steiner syndrome, *KMT2A*, phenotype, deep phenotyping, differential diagnosis, whole-exome sequencing

## Abstract

The condition known as 22q11.2 deletion syndrome (MIM #188400) is a rare disease with a highly variable clinical presentation including more than 180 features; specific guidelines for screening individuals have been used to support clinical suspicion before confirmatory tests by Brazil’s Craniofacial Project. Of the 2568 patients listed in the Brazilian Database on Craniofacial Anomalies, 43 individuals negative for the 22q11.2 deletion syndrome were further investigated through whole-exome sequencing. Three patients (6.7%) presented with heterozygous pathogenic variants in the *KMT2A* gene, including a novel variant (c.6158+1del) and two that had been previously reported (c.173dup and c.3241C>T); reverse phenotyping concluded that all three patients presented features of Wiedemann–Steiner syndrome, such as neurodevelopmental disorders and dysmorphic facial features (*n* = 3), hyperactivity and anxiety (*n* = 2), thick eyebrows and lower-limb hypertrichosis (*n* = 2), congenital heart disease (*n* = 1), short stature (*n* = 1), and velopharyngeal insufficiency (*n* = 2). Overlapping features between 22q11.2 deletion syndrome and Wiedemann–Steiner syndrome comprised neuropsychiatric disorders and dysmorphic characteristics involving the eyes and nose region; velopharyngeal insufficiency was seen in two patients and is an unreported finding in WDSTS. Therefore, we suggest that both conditions should be included in each other’s differential diagnoses.

## 1. Introduction

The condition known as 22q11.2 deletion syndrome (22q11.2DS) (MIM #188400) is a rare disease with autosomal dominant inheritance caused by heterozygous deletions of the 22q11.2 locus, usually occurring as de novo mutations. Its prevalence has been estimated to range from 1:3000 to 1:6000 live births, based on the diagnosis of infants with major birth defects and a few population screening studies conducted between the early 1990s and early 2000s using FISH technology, and it is considered the most frequent microdeletion in humans [1,2,3,4,5].

The most common features of 22q11.2DS include congenital heart disease, developmental delay, intellectual disability, palatal defects, immunodeficiency, endocrine abnormalities, and behavioral and psychiatric disorders. Nevertheless, the clinical picture is highly variable in presentation and severity and might include less frequent features in a list of more than 180 manifestations [2,3,5]. This remarkable clinical heterogeneity represents a diagnostic challenge, often delaying appropriate treatment and genetic counseling [3], and there is a broad range of differential diagnoses, including chromosomal abnormalities, monogenic conditions, polygenic disorders, and non-genetic causes. Genes or conditions of interest in the differential diagnosis of 22q11.2DS include *CHD7* (CHARGE syndrome), *DHCR7* (Smith–Lemli–Opitz syndrome), *JAG1* and *NOTCH2* (Alagille syndrome), *TBX1* (tetralogy of Fallot), other chromosomal disorders, VACTERL association, oculoauriculovertebral syndrome, and teratogenic exposures [6].

Due to the highly variable clinical presentation, several studies have been conducted with the aim of defining which patients would be eligible for screening; the applicability of the guidelines proposed by Monteiro et al. [3] was validated in 347 individuals, all investigated with fluorescent in situ hybridization (FISH) and/or multiplex ligation-dependent probe amplification (MLPA) tests for the deletion locus and further chromosomal microarray analysis (CMA) for the negative results. The study showed the high predictive value of congenital heart diseases and the significant association of hypernasal voice in 22q11.2DS as the most specific features [7]. The suggested clinical guidelines comprised absolute features (items A and B), core features (items C to G), and associated features (items H to N), described as follows:

A: Cardiac malformation with high predictive value, such as interruption of aortic arch type B, truncus arteriosus, and/or ventricular septal defect with pulmonary atresia (tetralogy of Fallot with pulmonary atresia).

B: Neonatal hypocalcemia secondary to idiopathic hypoparathyroidism.

C: Other conotruncal heart defects include classic tetralogy of Fallot, ventricular septal defect posterior malalignment, ventricular septal defects and subarterial or subpulmonary and/or aortic coarctation.

D: Palatal abnormalities such as velopharyngeal insufficiency, overt or submucous cleft palate, and/or cleft lip/palate.

E: Immunodeficiency confirmed by laboratory analysis and/or thymic hypoplasia/aplasia.

F: A typical face with four or more characteristic dysmorphisms, including at least three of the following: a long face, hooded eyelids, a tubular nose or other form of typical nose, or alar hypoplasia.

G: Schizophrenia.

H: Neurocognitive dysfunction such as a developmental delay, language developmental delay, and/or learning disability.

I: Cardiovascular abnormalities such as aortic arch alterations and/or pulmonary arterial tree alteration.

J: Two or more suggestive dysmorphisms in a patient aged 2 or older, OR one or more in a child younger than 2 years.

K: Hypernasal tone of voice.

L: Other cardiac defects include other types of ventricular septal defect, transposition of great arteries, a double right-outlet ventricle, an atrial septal defect, and/or patent ductus arteriosus.

M: Other palatal abnormalities include an isolated bifid uvula and/or a cleft lip.

N: Genitourinary malformations.

Indication for confirmatory testing is given when the patient fulfills any item from A or B, at least two items from C to G, one item from C to G plus at least two items from H to N, two or more items from H to N, or at least four items from H to N.

In nearly 85% of cases, 22q11.2 deletion comprises a 3 Mb interval (typical deleted region), while 10% of cases exhibit a proximal 1.5 Mb deletion, but atypical deletions might also be found [1,2,3]. The goal of the confirmatory tests is then to detect the deletion, and the most traditional methods are FISH and MLPA. Nowadays, chromosomal microarray analysis (CMA) and whole-exome sequencing (WES) have become widely used for these patients.

Brazil’s Craniofacial Project (BCFP) is a voluntary, multicenter, inter-institutional initiative and has developed strategies to perform diagnostic tests for 22q11.2DS in Brazil. Individuals and families are invited to have their clinical information recorded in the Brazilian Database on Craniofacial Anomalies (BDCA) and there are currently 2568 individuals with different diagnoses included in the database; BCFP then performs diagnostic evaluations at no cost to the family as part of research projects funded by public agencies [4,8].

Next-generation sequencing technologies such as whole-exome sequencing (WES) or whole-genome sequencing (WGS) have facilitated the reverse phenotyping approach in opposition to the phenotype-first approach [9]; that is, when the detection of a causative genetic abnormality conducts to a previously unobserved clinical phenotype indicating the diagnosis. Due to its high populational frequency, 22q11.2DS might also cooccur with other conditions, resulting in dual diagnosis [3,5]. Thus, the most recent tendency in clinical practice is to utilize a more broad approach combining different genomic tests for the detection of copy number variants and single-nucleotide variants in the same individual.

## 2. Materials and Methods

### 2.1. Selection and Consent of Research Subjects

A total of 2568 individuals were invited to join a research protocol approved by the Institutional Ethics Committee (CAAE 35316314.9.1001.5404 and 85020018.8.0000.5404) and were registered in the BDCA between 2006 and 2023. Written consent was obtained from the patients or legal guardians before procedures. Clinical geneticists examined all patients and fulfilled individual checklists based on the screening guidelines [3]. Cytogenetic and cytogenomic tests followed the clinical diagnosis, and individuals with negative results were investigated with WES for differential diagnosis. This manuscript presents and discusses the results of a subsample of 43 individuals registered in the BDCA with a suspected diagnosis of 22q11.2DS and negative cytogenomic results who were further investigated through WES, of which 3 (6.7%) had heterozygous pathogenic variants in the *KMT2A* gene. From now on, these three individuals will be mentioned as patients 1, 2, and 3. The other positive results will be discussed at another appropriate moment.

### 2.2. Cytogenetic and Cytogenomic Analysis

For 22q11.2 deletion identification, initially, chromosomal analysis involving the G-banding technique was conducted according to standard protocols. Over the years, different methods were incorporated into the genetic workflow of the laboratory routine to investigate the 22q11.2 deletion. The FISH technique was performed using the TUPLE1 probe (Kreatech Diagnostics, Amsterdam, the Netherlands), MLPA was performed according to the manufacturer’s instructions with the P250-B2 kit (MRC-Holland, Amsterdam, the Netherlands), and CMA was performed using different platforms. For patients 1 and 3, CMA was performed using the Cytoscan HD (Thermo Fisher Scientific, Inc.—Life Technologies, Carlsbad, CA, USA) chip and analyzed with the software Chromosome Analysis Suite ChAS version 3.3.0.139 (r10838)—Affymetrix®, Santa Clara, CA, USA) (hg19); data were compared with 380 control individuals (HapMap; BioServe Biotechnologies, Hyderabad, India). For patient 2, low-pass whole-genome sequencing with 1× coverage for CNVs detection was performed using the NovaSeq 6000 S4 Reagent Kit v1.5 and the NovaSeq 6000 equipment (Illumina, San Diego, CA, USA) and analyzed with N×Clinical software version 4.0 (Biodiscovery, El Segundo, CA, USA).

### 2.3. Whole-Exome Sequencing Analysis

For 43 patients with negative cytogenomic results for the presence of the 22q11.2 deletion, WES was performed by the 3billion Company using a NovaSeq 6000 platform (Illumina, San Diego, CA, USA) and analyzed with xGen Exome Research Panel v2 (Integrated DNA Technologies, Coralville, IA, USA) for one part of the cohort, including patients 1 and 2. Alternatively, WES was performed with the Agilent SureSelect Target Enrichment vs. (Agilent Technologies, Santa Clara, CA, USA) in an Illumina HiSeq platform (Illumina, San Diego, CA, USA) for another part of the cohort, including patient 3. For both WES methods, variant classification according to the American College of Medical Genetics and nomenclature followed the recommendations of the Human Genome Variation Society [10,11].

### 2.4. Sanger Sequencing Analysis

For patient 1 and her parents, Sanger sequencing was performed to investigate the inheritance of the variant using the BigDye^®^ Terminator v.3.1 Cycle Sequencing kit (Applied Biosystems) and the SeqStudio^TM^ Genetic Analyzer (ThermoFisher Scientific, Applied Biosystems, Waltham, MA, USA). Chromatograms were analyzed with the Chromas software (https://technelysium.com.au/wp/chromas/), accessed on 29 June 2023.

## 3. Results

A total of 3 (6.7%) of the 43 patients who were negative for 22q11.2DS and further investigated through WES had heterozygous pathogenic variants in the *KMT2A* gene.

### 3.1. Patients’ Descriptions

Patient 1 is a female who was first seen at the age of 11 years and referred for clinical genetic evaluation due to a learning disability and a probable diagnosis of congenital adrenal hyperplasia. She is the second child of a nonconsanguineous couple; the father was 40, and the mother was 33 at conception. The pregnancy was uneventful, and the delivery was at term by cesarean section due to fetal bradycardia; her weight was 2750 g (−1.01 SD) and her length was 45 cm (−2.14 SD). Her development was within the normal range except for a language delay, and she presented two episodes of seizures at the age of 30 months. The initial assessment revealed a weight of 42.8 kg (+0.25 SD), a stature of 145.4 cm (−1.62 SD), and a head circumference of 51.4 cm (−0.74 SD); the dysmorphological findings comprised a long face, low-set ears with hypoplastic ear lobes, thick eyebrows with lateral mild madarosis, bilateral blepharoptosis, narrow palpebral fissures, a broad nasal root, a bulbous nasal tip, a short philtrum, a high palate, downturned mouth corners, retrognathia, dry skin, and lower-limb hypertrichosis. Complementary investigations included an abdominal ultrasound, brain MRI, determination of bone age, audiometry evaluation, and fundoscopy, which were all within normal parameters. An EEG showed epileptiform discharges. Target gene sequencing of *CYP21A2* was negative for deleterious variants. A fibroscopy examination of the entire upper airway revealed an asymmetric soft palate closure, indicating a velopharyngeal insufficiency. At the last evaluation, at the age of 19, her final stature was 146.5 cm (−2.62 SD), her head circumference was 53 cm (+0.13 SD), and her weight was 56 kg (−0.07 SD). She fulfilled clinical criteria D, H, and J of the guidelines for 22q11.2DS [3].

Patient 2 is a female who was referred for genetic evaluation at the age of three years due to congenital heart disease, dysmorphic features, and behavior problems. She is the third child of a nonconsanguineous couple; the father was 29 and the mother was 31 at conception. The pregnancy was complicated by a urinary infection, and delivery was at term by cesarean section due to fetal distress and meconium-stained amniotic fluid; her weight was 3710 g (+1.47 SD), her length was 48 cm (−0.41 SD), and her head circumference 35.5 cm (+1.78 SD). Her neurological development was borderline, and she presented four episodes of pneumonia in infancy. The initial assessment revealed a weight of 11.5 kg (−1.89 SD) and a stature of 86 cm (−2.97 SD); dysmorphic findings comprised frontal bossing, thick eyebrows, epicanthal folds, blepharoptosis, up-slanting and narrow palpebral fissures, strabismus, a broad nasal root, narrow nostrils, a smooth and long philtrum, downturned mouth corners, dental malocclusion, retrognathia, a heart murmur, fifth-finger clinodactyly, lower-limb hypertrichosis, aggression, and agitation. The echocardiogram revealed a double atrial and ventricular septal defect associated with persistent foramen ovale, and the IQ test results (WISC-III) were compatible with mild intellectual deficiency. Episodes of self-harm and hetero-aggression complicated her learning disability, and at the age of 13, she presented with auditory hallucinations, resulting in a diagnosis of schizophrenia managed with carbamazepine, chlorpromazine, promethazine, clomipramine, haloperidol, quetiapine, and diazepam. At age 17, her weight was 70.4 kg (+1.41 SD), her stature was 152.5 cm (−1.64 SD), and her head circumference was 54 cm (−0.35 SD). She fulfilled clinical criteria G, H, and L of the guidelines for 22q11.2DS [3].

Patient 3 is a female who was referred for genetic evaluation at the age of three months due to facial dysmorphisms. She is the only child of a nonconsanguineous couple; the father was 25 and the mother was 18 at conception. The pregnancy was uneventful, and the delivery was at term by cesarean section due to abnormal progression of labor; her weight was 2850 g (−0.74 SD), her length was 45 cm (−2.14 SD), and her head circumference was 35.5 cm (+1.78 SD). She presented muscle hypotonia, and her global development was severely impaired. Dysmorphic findings comprised macrocephaly, low-set ears, epicanthal folds, down-slanting palpebral fissures, a bulbous nasal tip, a high and arched palate, retrognathia, a short neck, diastasis recti, fovea coccygea, brachydactyly on the hands, prominent calcaneus, and plantar creases. Her medical comorbidities include velopharyngeal insufficiency and seizures from the age of 23 years. She also exhibits anxiety, agitation, self-harm, and hetero-aggression, which are currently being managed with oxcarbazepine and escitalopram. Anthropometric data at 26 years showed a weight of 81 kg (+2.17 SD), a stature of 157 cm (−0.94 SD), and a head circumference of 58.5 cm (+2.32 SD). She fulfilled clinical criteria D, H, J, and K of the guidelines for 22q11.2DS [3].

### 3.2. Cytogenetic and Cytogenomic Results

Routine karyotyping resulted as normal for all three patients. MLPA was performed in all three patients, and patient 3 was also submitted to FISH analysis; all three patients were negative for deletion at the 22q11.2 locus. Low-pass whole-genome sequencing was normal for patient 2, and in patient 3, the CMA result showed a duplication of 191 kb at 7q11.22—arr[GRCh37] 7q11.22(71889833_72081552) × 3, classified as a variant of uncertain significance.

### 3.3. Molecular Results

The three patients presented heterozygous pathogenic variants in the *KMT2A* gene. Patient 1 showed a variant at a canonical splicing site, while patients 2 and 3 showed variants that resulted in a premature stop codon. The molecular results of the three patients are shown in Table 1.

**Figure 1:**
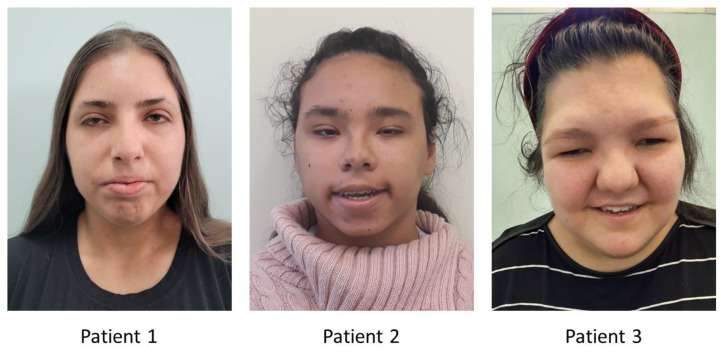
Craniofacial features of the three patients with *KMT2A* variants, respectively, at 19, 17, and 26 years.

Figure 1 shows the facial features of the three patients at the most recent clinical evaluation. The clinical findings are summarized in Table 2.

## 4. Discussion

All three participants met the inclusion criteria and presented several clinical findings listed in the guidelines for 22q11.2DS [3]. The common feature in all three cases was neurodevelopmental abnormalities; velopharyngeal insufficiency was detected in two patients and congenital heart disease in one.

Developmental delay in 22q11.2DS is an almost constant finding, and learning disabilities are widespread in those with the condition, although intellectual disability is estimated to occur in less than 50% of cases, and usually to a mild degree. Hyperactivity and attention deficit disorder, anxiety, obsessive–compulsive behavior, and bipolar disorder are frequently found in both children and adults. Schizophrenia and other schizoaffective disorders are reported to affect 10% to 30% of individuals [3], which reinforced the diagnostic suspicion in patient 2.

Among the palatal abnormalities in 22q11.2DS, velopharyngeal insufficiency is the most reported in 29% to 50% of all cases [3], which was a key feature for diagnostic suspicion in patients 1 and 3.

Despite this, all three patients had negative results for 22q11.2DS and presented with deleterious variants in the *KMT2A* gene. The c.6158 + 1del variant in patient 1 is novel. The c.173dup[p.(Ala59Glyfs*88)] variant in patient 2 has been previously reported [14], as has the c.3241C>T[p.(Arg1081*)] variant in patient 3 [15].

Somatic mutations and rearrangements in the *KMT2A* gene have been described in tumors, mainly blood malignancies, and translocations involving the *KMT2A* gene are common in different types of leukemia [16]. Still, Wiedemann–Steiner syndrome (WDSTS; MIM #605130) is currently the only dysmorphic phenotype associated with germinal mutations in the *KMT2A* gene [17]. The *KMT2* gene family (histone-lysine N-methyltransferase 2) corresponds to a group of genes responsible for encoding methyltransferases that carry out mono-, di-, or trimethylation of lysine 4 of histone 3 (H3K4) [18]. It is essential for the process of hematopoiesis and neurodevelopment, and also contributes to the regulation of genes such as the *HOX* and *WNT* gene families, which result in transcription factors and important signaling pathways during the beginning of normal embryo development [17].

In 1989, Wiedemann et al. reported a single male patient presenting with pre- and postnatal growth deficiency, developmental delay, and craniofacial dysmorphisms, suggesting that the clinical picture could represent a new syndrome [19]. Steiner and Marques reported a girl with similar features and limb hypertrichosis, mainly cubital [20]. A few reports arose in the literature in the following years until this combination of features was named Wiedemann–Steiner syndrome [21]. After the discovery of de novo heterozygous variants in the *MLL* (*KMT2A*) gene [22], a molecular marker was available to confirm further cases and expand the phenotypic spectrum of the disorder [12,23].

WDSTS is now known to be a rare autosomal dominant disorder caused by mutations in the *KMT2A* gene. Still, recently, a de novo mutation in the *ADAMTS8* gene was reported in a girl with clinical features similar to WDSTS [24]. The differential diagnosis list for WDSTS includes several other chromatinopathies such as Coffin–Siris syndrome, Rubinstein–Taybi syndrome, Kabuki syndrome, Cornelia de Lange syndrome, Noonan syndrome, and chromosomal abnormalities [12,17,25]. Some syndromes, such as Kabuki and Cornelia de Lange, also show phenotypic overlap with 22q11.2DS [26], indicating a relation between 22q11.2DS and WDSTS phenotypes. This is the first report focusing on the differential diagnosis of WDSTS and 22q11.2DS (Table 1).

WDSTS is a clinically recognizable neurodevelopmental disorder syndrome characterized by variable degrees of language or global developmental delay, intellectual deficiency, behavioral problems, pre- and/or postnatal growth deficiency, hirsutism (back and limbs, mainly cubital) with thick eyebrows and long eyelashes, and facial dysmorphisms. The distinctive or “typical” face includes hypertelorism, down-slanting and narrow palpebral fissures, a broad nasal tip, a long philtrum, and a thin upper vermilion border. Skeletal and visceral features have also been described in some instances, including congenital heart disease and kidney, ophthalmologic, dental, and endocrine abnormalities [12,17,25]. It is a multisystemic condition with a highly variable presentation, and the combination of typical face and hypertrichosis cubiti, initially considered a key feature, is not universal.

Thus, although none of the present reported subjects had the typical facies, reverse phenotyping concluded that all three cases filled out features of WDSTS: short stature was a finding in patient 1, lower limb hypertrichosis was seen in patients 1 and 2, and variable degrees of developmental delay and facial dysmorphisms were present in all three, compatible with the diagnosis of WDSTS spectrum.

WDSTS is usually associated with pre- and/or postnatal growth deficiency. In the large cohort of Sheppard et al. [12], few individuals had anthropometrics greater than the 95th percentile for weight (5.3%), height (1.0%), and head circumference (3.7%). The individual with a height greater than the 95th percentile had a p.Ala1030Glyfs*9 variant; the individuals with a head circumference greater than the 95th percentile had the p.Arg1067Trp, p.Lis1751*, and p.1219Valfs*2 variants. Patient 3 has macrocephaly, which was also described in patient 7 by Li et al. [15], who presented a p.Ser774Valfs*12 variant.

There was a wide range of ophthalmologic abnormalities in the cohort of Sheppard et al. [12]. The most common was strabismus in 37.5% of individuals, and surgical correction of blepharoptosis was performed in three individuals. In the present sample, blepharoptosis was found in two patients and strabismus in one.

Patient 2 presented with an atrial and ventricular septal defect associated with persistent foramen ovale. Structural cardiac defects are usually described in 22q11.2DS and were seen in 28.4% of the individuals with WDSTS, usually characterized by minor anomalies [23]; however, arrhythmia was seen in two participants, one requiring a pacemaker [12]. Congenital heart diseases were more significant in the French than in the Chinese patients with WDSTS [15,22].

Hypernasal speech and submucous or overt cleft palate, common features of 22q11.2DS, have been previously described in WDSTS, and Caucasian individuals were significantly more likely to have bifid uvula [12,27,28], but velopharyngeal insufficiency as seen in patients 1 and 3 is an unreported finding in WDSTS.

Concerning the molecular results reported herein, novel variants in the *KMT2A* gene are constantly described in anecdotal reports or series of cases, but recurrent variants are becoming frequent as the number of published cases of individuals with WDSTS individuals.

The variant in patient 2 was described by Feldman et al. [14] in a 2-year-old boy with growth restriction, oligohydramnios, significant developmental delay, mild microcephaly, plagiocephaly, upturned nose, and neck pterygia; no abnormalities in the extremities or hypertrichosis were noted. No photographs of this individual were available, and besides severe neurological features, his clinical picture seems to differ from that of patient 2. We have also observed the same variant in a boy presenting with facial dysmorphisms, especially severe bilateral ptosis requiring surgical correction, without major neuropsychiatric features.

Patient 3 has the previously reported variant p.Arg1081* described in patient 6 in the cohort of Li et al. [15]. The patient was also a girl with thick hair and eyebrows, long eyelashes, hypertrichosis cubiti and in the back, and a high palate. She did not present neuropsychiatric findings. Once more, the clinical presentation is diverse in both individuals.

Loss-of-function variants were more likely to present with hypotonia, while non-loss-of-function variants were more likely to present with seizures [12], but the lack of a genotype–phenotype seems to occur in WDSTS [12,22,25].

## 5. Conclusions

Finally, comparing the frequency of the clinical features to the data from a large cohort of 104 individuals diagnosed with WDSTS [12] and a combined cohort of up to 906 individuals with 22q11.2DS [1,2], an overlap of features was observed among both conditions, despite their difference in frequencies. This overlap particularly concerns neuropsychiatric disorders, exemplified as developmental delay, intellectual deficiency, and attention deficit hyperactivity disorder. There are also some dysmorphic characteristics involving the eyes and nose region, such as thick eyebrows, long eyelashes, narrow palpebral fissures, blepharoptosis, and a bulbous nose tip, and this report presents velopharyngeal insufficiency as a new finding in WDSTS. On the other hand, hair abnormalities such as hypertrichosis and thick eyebrows are typically described in WDSTS, while congenital heart diseases are far more associated with 22q11.2DS than with WDSTS. Thus, we suggest that WDSTS and 22q11.2DS should be included in each other’s differential diagnoses.

## Figures and Tables

**Table 1:** Molecular results of the three patients and classification following ACMG criteria.

Patient	Variant	ACMG Criteria [10]	Classification
1	NM_001197104.2(*KMT2A*):c.6158+1del;p.?	PVS1 + PM2 + PM6	pathogenic
2	NM_001197104.2(*KMT2A*):c.173dup;p.(Ala59Glyfs*88)	PVS1 + PM2 + PP5	pathogenic
3	NM_001197104.2(*KMT2A*):c.3241C>T;p.(Arg1081*)	PVS1 + PM2 + PP5	pathogenic

**Table 2:** Clinical findings of the three patients compared to the frequency of features described in Wiedemann–Steiner syndrome (WDSTS) [12] and in 22q11.2DS [1,2].

Clinical Finding		Patient 1	Patient 2	Patient 3	WDSTS	22q11.2DS
Growth	Short stature	+	-	-	57.4%	15%
Craniofacial	Macrocephaly	-	-	+	3.7%	+
	Microcephaly	-	-	-	34.6%	10%
	Long face	+	-	-	nd	+
	Malar flattening	+	+	-	nd	+
	Down-slanting palpebral fissures	+	-	+	49.5%	nd
	Narrow palpebral fissures	+	+	-	69.3%	+
	Blepharoptosis	+	+	-	43.0%	4%
	Broad nasal root	-	+	-	63.4%	+
	Bulbous nasal tip	+	-	+	63.6%	60%
	Low-set ears	+	-	+	nd	13%
	Retrognathia	+	+	-	nd	21%
	Velopharyngeal insufficiency	+	-	+	nd	42%
Skin and hair	Thick eyebrows	+	+	-	75.5%	nd
	Long eyelashes	+	-	-	71.3%	nd
	Hypertrichosis cubiti	-	-	-	57.0%	nd
	Hypertrichosis (other areas)	+	+	-	45.4–67.3% ^1^	nd
	Dry skin	+	-	-	nd	nd
Behavior	ADHD	-	+	+	44.3%	54%
	Anxiety	-	+	+	65% ^2^	8%
	Schizophrenia	-	+	-	nd	~25%
Neurological	DD/ID	+	+	+	97.0%	30%
	Seizures	+	-	-	20.0%	+
Other	Eye abnormalities ^3^	+	+	-	20.5–37.5%	4–49%
	ASD/VSD/PFO	-	+	-	nd	21%
	Brachydactyly	+	-	+	+	nd
	Fifth-finger clynodactyly	-	+	-	+	nd

Key: + = present; - = absent; nd = no data; ADHD = attention deficit hyperactivity disorder; ASD = atrial septal defect; DD/ID = developmental delay/intellectual deficiency; PFO = persistent foramen ovale; VSD = ventricular septal defect; ^1^ lower limbs and back, respectively; ^2^ data from Ng et al. [13]; ^3^ excluding blepharoptosis.

## Data Availability

The data supporting this study’s findings are available from the corresponding author upon reasonable request.
